# Are weight control and food waste a trade-off?: A clustering of pre-meal portion planning and plate-clearing behaviors among Japanese adult consumers

**DOI:** 10.1007/s00394-025-03837-0

**Published:** 2025-11-18

**Authors:** Yui Kawasaki, Sayaka Nagao-Sato, Misa Shimpo, Rie Akamatsu

**Affiliations:** 1 Institute for SDGs Promotion, Organization for Social Implementation of Sustainability, Ochanomizu University, 2-1-1 Otsuka Bunkyo-ku, Tokyo, 112-8610 Japan; 2 Graduate School of Public Health, Shizuoka Graduate University of Public Health, 4-27-2, Kita Ando, Aoi-ku, Shizuoka, 420-0881 Japan; 3 Department of Food and Health Sciences, Faculty of Health and Human Development, The University of Nagano, 8-49-7, Miwa, Nagano city, Nagano 380-8525 Japan; 4 Natural Science Division, Faculty of Core Research, Ochanomizu University, 2-1-1 Otsuka Bunkyo-ku, Tokyo, 112-8610 Japan

**Keywords:** Food waste, Weight control, Plate-clearing behaviors, Behavioral pattern, Consumer, Pre-meal portion planning

## Abstract

**Purpose:**

Plate-clearing behavior (PCB), a maladaptive behavior in a food environment characterized by large, energy-dense portions of food, may contribute to weight gain over time. If a portion size is selected that causes overeating, a trade-off exists between food waste and weight gain due to PCB. However, patterns between PCBs and pre-meal portion planning to prevent overeating (PPP-O) remain overlooked. This study aimed to identify these patterns in various meal situations and describe the demographic, anthropometric, psychological, and lifestyle-related characteristics of those following each pattern.

**Methods:**

Overall, 1,707 Japanese participants responded to a web-based anonymous questionnaire in February 2023. Cluster analysis was performed to identify patterns in PPP-O and PCB. Multiple logistic regression analysis was used on clusters of participant characteristic variables.

**Results:**

The median age of the participants was 40 (25th and 75th percentile: 30, 50) years (female =  865, 50.7%). Four clusters with independent predictors were identified: low PPP-O and high PCB, moderate PPP-O and high PCB, moderate PPP-O and low PCB, and high PPP-O and low PCB. Low BMI and a higher positive attitude toward food waste were independent predictors in high PPP-O and low PCB, while high BMI was an independent predictor in moderate PPP-O and high PCBs and low attitude toward food waste were independent predictors in moderate PPP-O and low PCB. Psychological factors were described as independent predictors of several clusters.

**Conclusion:**

The results support the possibility of a trade-off between food waste and weight gain if PPP-O is not implemented.

**Supplementary Information:**

The online version contains supplementary material available at 10.1007/s00394-025-03837-0.

## Introduction

Obesity is prevalent in many countries and is recognized as a major public health problem. Researchers investigating plate-clearing behavior (PCB) in individuals in the UK and US have identified that it contributes to consuming more food, especially from larger portions [1]. In a food environment characterized by large, energy-dense portions of food, plate-clearing is a maladaptive behavior that could contribute to weight gain over time [1–4]. A significantly higher proportion of people with obesity has been reported among those with plate-clearing tendencies, and this association remained significant after adjusting for the effect of portion size [1–4]. Moreover, Sheen et al. have reported that individual concerns regarding food waste are associated with plate-clearing tendencies [1, 2].

Food waste increases environmental impacts and adversely affects global environment and health, causing greenhouse gas emissions, burdening waste management systems, and increasing hunger and malnutrition owing to population growth and food distribution imbalances [5, 6]. However, the authors’ previous study findings on Japanese participants differ from those of the aforementioned studies: Japanese adult consumers’ Body Mass Indexes (BMIs) are not correlated with food waste avoidance behaviors, including PCBs [7]. Considering that the Japanese population faces a more significant issue with thinness [8], particularly among women of all ages, rather than obesity, and that more of its citizens generally focus on preventing weight gain compared to those in the UK, it is expected that the Japanese population balances efforts to avoid food waste with strategies for managing weight before and after their meals [9].

Pre-meal portion planning to prevent overeating (PPP-O) have been overlooked in previous studies examining the relationship between PCBs, body weight, and food waste concerns [1–4, 7]. While there is no academic definition for PPP-O, this study describes it as the behavior of determining portion sizes before a meal to prevent overeating in various real-world settings when someone recognizes that more food is likely to be chosen than they can reasonably eat. PPP-O can be considered a form of dietary restriction. When the objective is to limit energy intake to prevent excessive energy consumption, pre-meal planning inherently incorporates an element of restriction. There is some scale to measure dietary restriction behaviors encompass actions related to PPP-O (Stunkard & Messick [42]). PPP-O is exemplified by the decision to order a larger portion than one can comfortably consume at a restaurant, contrasted with the choice of a smaller portion when the menu offers a variety of portion sizes. The previous study have shown that in settings where individuals can self-determine their portion sizes, they do so according to their expected satiety [10], and that planning meals in advance is the strongest indicator of how much they will consume [11]. Therefore, if PPP-O is implemented in the real-setting, such as at home (home cooking and/or eating prepared foods) and at restaurants, individuals’ body weight may not increase without leaving leftovers; however, if a portion size is selected that causes overeating, there is a trade-off between food waste and weight gain due to PCB. Numerous studies have shown that controlling portion sizes can prevent weight gain and/or maintain individuals’ weights [12, 13].

However, the effect of a combination of PCBs and PPP-O on body weight has not yet been examined. For example, while the implementation of both may be associated with better health and environmental behaviors, to date, evidence of better weight control is lacking. There may be differences in weight control and psychological status depending on the degrees of PPP-O and PCBs implementation. Although previous studies have reported that people with stronger food waste concerns are more likely to implement PCBs [2], little has been done to examine health awareness, which is expected to affect PCBs and PPP-O, as well as food waste concerns. Lifestyle-related factors, such as the frequency of eating out, ready-made meal consumption, and cooking, may also influence behavioral patterns, as they relate to the frequency of PPP-O and PCB implementation. According to Japanese statistics that calculate annual changes in the percentage of food expenditures accounted for by eating out, ready-made meal, and fresh food, eating out has remained flat, but ready-made meal has increased year by year; fresh food, on the contrary, has been declining [14]. PPP-O and PCBs will likely be implemented at any eating occasion, including in different situations (eating at home or in restaurants) or with different meals (home cooked or pre-prepared food). However, research on these behaviors in these scenarios is scarce.

Moreover, Japanese people have unique norms regarding food waste avoidance behaviors, such as eating what you are served without leaving any leftovers, following Buddhism and Shintoism [7, 15]. These norms are based on Japanese “Gratitude for Food” [7, 15]. In Japan, definition of gratitude for food originated from the Buddhist and Shinto belief that all things have life in them [7, 16]. Nowadays, the object of gratitude for food has expanded beyond the food itself to include the producers and cooks [16, 17]. Parents and educators teach that food is the same as life, and that gratitude for food is synonymous with gratitude for life [7, 16, 17]. The Shokuiku Basic Law, a law enacted to promote dietary education for children, also recommends gratitude for food and clearing dishes [18].

Exploratory data-driven methods, such as cluster analysis, can be used to gain insight into behavioral patterns [19]. By identifying the patterns of PPP-O and PCBs in various dietary behavioral scenarios and describing the demographic, psychological, and lifestyle characteristics of those who follow each pattern, it is possible to make policy recommendations to promote food waste reduction while aiming to maintain individuals’ healthy body weights. Therefore, this study aims to (1) identify patterns of PPP-O and PCBs in various meal situations and (2) describe the demographic, anthropometric, psychological, and lifestyle-related characteristics of those who follow each pattern.

## Methods

### Study design

This study was part of a larger longitudinal online survey that aimed to examine the association between PPP-O, PCBs, and weight control in 2023. The detailed data collection process is reported in a previous study [16]. Baseline data of 1,800 men and women aged 18–59 years living in Japan were used in the present study. In addition, to examine the test–retest reliability and criterion validity of the original items in this study, we used data from 1,380 individuals who responded to a follow-up survey conducted one week after the baseline survey [16].

### Variables

Participants’ demographic, anthropometric, psychological, and lifestyle-related data, as well as PPP-O, PCBs, and recognizing the need to control portion sizes, were used as independent variables in the present study. Detailed question items are shown in Appendix A.

#### Pre-meal portion planning to prevent overeating

To measure PPP-O, eight questions were developed. Each item was divided into eating scenes, such as eating out and at home. With respect to PPP-O, five items concerning the eating-out scenario (PPE-1–5; Table 1) were developed based on the present study to describe the characteristics of people who order the portion-controlled food at restaurants [20]. Three items concerning the home scenario (PPH-1–3) were developed considering the food choice scenario listed in a previous study [21]. A 6-point Likert scale was used (1 =  not at all, 6 =  always). Criterion validity of these items was confirmed by the correlation coefficients of each item with “eating selected foods without leaving any leftovers,” which is one of the sustainable and healthy dietary behaviors developed in a previous study (ρ =  − 0.13 to − 0.20, *p* <  0.001) [7]. Repeat reliability was also tested using the same items asked in a one-week follow-up survey (Spearman’s correlation coefficient: ρ =  0.45–0.66,* p* <  0.001 for each item).

**Table 1:** Items of questions developed for the survey

Items of questions by each theme	Options
*Pre-meal portion planning to prevent overeating*^*a*^	1: Not at all–6: always
< Eating out >	
PPE-1. I check the serving size of food before entering a restaurant or choose a restaurant where I already know the serving size of food or can adjust the serving size of food.	
PPE-2. When I select a menu, I check weather I can eat the food I want to order without difficulty.	
PPE-3. When I want to order food with a larger serving size than I can eat without difficulty, I choose a smaller size if the menu has a range of sizes.	
PPE-4. When I want to order food with a larger serving size than I can eat without difficulty, I order food with a smaller serving size if available and if I can get a discount by reducing the size.	
PPE-5. When I want to order food with a larger serving size than I can eat without difficulty, I ask the waiter for a smaller portion even if the menu does not have a size range.	
< Home >	
PPH-1. When I feel that the amount of food I prepare is more than I can eat without difficulty, I reduce it to the amount I can eat.	
PPH-2. When I feel that the amount of food (e.g. lunch boxes, prepared foods, or instant foods) I purchased is more than I can eat without difficulty, I reduce the amount until I can finish the food.	
PPH-3. When the amount of food prepared by family members is more than I can eat without difficulty, I reduce the amount until I can finish the food.	
*Plate clearing behavior*^*b*^	
< Eating out >	
PCE-1. When eating out, if you feel that the amount of food is too much (there is more food in front of you than you can eat without difficulty), do you finish the meal, even if you have to push yourself?	1: I do not eat (leave/take home) more than I can eat without difficulty on the spot, regardless of serving size of the food – 5: I eat more than twice as much food as I can eat without difficulty
< Home >	1: I do not eat (leave/take home) more than I can eat without difficulty on the spot, regardless of how much the menu offers – 5: I eat more than twice as much food as I can eat without difficulty
PCH-1. When I cook and prepare (serve) a larger amount of food than I can eat without difficulty, …	
PCH-2. When I feel that the amount of food I purchased (e.g. lunchbox and prepared food) or instant food I cooked is more than I can eat without difficulty, …	
PCH-3. When the amount of food prepared by my family members was more than I could reasonably eat, …	
*Recognition of the need to control portion size*	1: Not at all–6: always
1. When I eat out, I sometimes feel like ordering a menu item with a larger portion size than I can comfortably eat.	
2. When cooking, I sometimes feel that the amount of food prepared is more than I (we) can comfortably eat.	
3. I sometimes feel that the amount of prepared foods that I buy is more than I can comfortably eat.	
4. I sometimes feel that the amount of food prepared by my family is more than I can comfortably eat.	

^*a*^ Criterion validity of these items was confirmed by the correlation coefficients of each item with “eating selected foods without leaving any leftovers,” which is one of the sustainable and healthy dietary behaviors developed in a previous study (ρ =  − 0.13 to − 0.20, *p* <  0.001) [7]. Repeat reliability was also tested using the same items asked in a one-week follow-up survey (Spearman’s correlation coefficient: ρ =  0.45–0.66,* p* <  0.001 for each item).

^*b*^ The criterion validity of these items was confirmed by the correlation coefficients of each item with hunger and satiety cues (three items, 4-point Likert scale; Cronbach’s alpha  =  0.657), a subscale of the expanded mindful eating scale developed in a previous study (ρ =  − 0.24 to − 0.31, *p* <  0.001) [22]. Test–retest reliability was also confirmed using the same items (PE-1 and PH-1–3) in the one-week follow-up survey (Spearman’s correlation coefficients: ρ =  0.68–0.74, *p* <  0.001 for each item)

#### Plate-clearing behaviors

Eight original question items were developed for PCBs (Table 1). The PCB scale, developed in a previous study, was not used in the present study [4]. This is because, culturally, the Japanese have a strong norm warning people to “not leave food uneaten” [7, 15]. The authors’ previous research with Japanese participants found response bias and ceiling effects in many of the items regarding “eating without leaving food” [16], making it difficult to accurately measure PCBs with the questionnaire items developed for a Western context. These items were designed to ask about PCB in each dietary behavior situation, focusing on “what size food portion would you be willing to force yourself to eat?” based on the assumption that the participants follow the norm of eating without leaving any leftovers. Participants were asked whether they eat all foods that they cannot eat with difficulty in four food choice scenarios: eating out (PCE-1), home cooking (PCH-1), eating prepared food (PCH-2), and eating food prepared by their family member (PCH-3) [21]. The criterion validity of these items was confirmed by the correlation coefficients of each item with hunger and satiety cues (three items, 4-point Likert scale; α = 0.66), a subscale of the expanded mindful eating scale developed in a previous study (ρ = − 0.24 to −  0.31, *p* <  0.001) [22]. Test–retest reliability was also confirmed using the same items (PCE-1 and PCH-1–3) in the one-week follow-up survey (Spearman’s correlation coefficients: ρ =  0.68–0.74, *p* <  0.001 for each item).

#### Recognition of the need to control portion sizes

Additionally, to consider interpersonal variability in the frequency of the need to control portion sizes, four items were developed based on the aforementioned four scenarios, such as eating out, home cooking, eating prepared food, and eating food prepared by family members (1 =  not at all, 6 =  always; Table 1).

#### Psychological variables

Interest in health, attitudes toward avoiding food waste, and gratitude for food were included in the psychological data. The Interest in Health Scale, which was developed for Japanese adults and comprises a 12-item scale with three factors, was used (α = 0.88) [23]. Respondents answered using a 4-point Likert scale (1 = strongly disagree, 4 =  strongly agree). With respect to attitudes toward food waste, three items used in previous study were selected to measure participants’ attitudes [24]. A 6-point Likert scale (1 =  strongly disagree, 6 = strongly agree) was used in this study (α = 0.84). In addition, gratitude for food was measured using the Gratitude for Food Scale for Adults (GFS-A) [16]. This scale comprises one factor and five items scored on a 4-point Likert scale (1 =  strongly disagree, 4 = strongly agree; α = 0.92).

#### Lifestyle-related variables

Lifestyle-related variables included eating habits and physical activity. Regarding eating habits, the participants were asked about their frequency of eating out, preparing meals, eating together, and cooking, while considering items to measure PPP-O and PCBs. A 6-point Likert scale was used (1 = once in a month or less, 6 = twice a day or more), except for cooking frequency (1 = not at all, 8 =  three times a day or more). Moreover, the short form of the International Physical Activity Questionnaire (IPAQ) was adopted. This scale examines the duration and frequency of three types of activities: walking, moderate-intensity activity, and vigorous-intensity activity [25].

#### Demographic and anthropometric data

Demographic and anthropometric data included the participants’ ages, sex (1 = male, 2 = female), height, weight, living status (living alone or with others [one-, two-, three-, or four-generation family]), education (1 = elementary or junior high school to 4 = college, university, or graduate school), and household income (1 =  <  2,000,000 to 6 = >  10,000,000 JPY/year). Height and weight were recorded in centimeters and kilograms, respectively (integral numbers).

### Analysis

In the present study, the hypotheses and analytic plan were specified before the data were collected; furthermore, any data-driven analyses are clearly identified and discussed appropriately. Since the questions to measure PPP-O and PCBs used in this study were created assuming a situation in which there is more food in front of oneself than one can comfortably eat, it is possible that data from participants are not aware of such situations undermines the validity of the study may be obtained. Therefore, 93 participants who answered 1 =  “not at all” to all four questions concerning the frequency of recognizing the need to control portion sizes were excluded from baseline data (n =  1,800). Finally, 1,707 (baseline) and 1,338 (1-week follow-up) participants were included in the analysis.

The BMI of each participant was calculated based on their height and weight. The total scores for each psychological variable (interest in health, attitude toward recognizing the need to control portion sizes, avoiding food waste, and gratitude for food) were calculated. Total scores of interests in health (ranging from 12 to 48), attitude toward avoiding food waste (3–18), and the GFS-A (5–20), were calculated. Participants were categorized into low, moderate, and high physical activity levels, and the total metabolic equivalents in minutes per week were calculated based on the guidelines of the IPAQ and according to their responses.

The Shapiro–Wilk test indicated that the samples were not normally distributed (*p* <  0.05); therefore, non-parametric analysis was performed. Data are presented as medians and 25th and 75th percentiles. A cluster analysis was performed to identify patterns of PPP-O and PCBs implementation, and 12 items related to PPP-O and PCBs were incorporated as indicators. A two-step cluster analysis was applied to establish cluster groups. In the first step, small clusters were created based on distance, as in K-means. Next, the small clusters were combined in a stepwise manner, as in hierarchical cluster analysis. The fit of the model was determined using Schwarz’s Bayesian information criterion (BIC) and the average silhouette coefficient.

To describe participants’ characteristics of the identified cluster groups, the mean frequencies of each item in relation to PPP-O and PCBs in each cluster were described. Kruskal–Wallis and chi-square tests were used to describe participants’ backgrounds, such as their demographic, anthropometric, psychological, and lifestyle-related factors, by each cluster.

Multiple logistic regression analysis with a stepwise method was used to calculate the odds ratios (ORs) and 95% confidence indices (CIs) assigned to clusters based on the background variables. In this analysis, four multiple logistic regression analyses with clusters as the dependent variable were performed. For example, in the logistic regression analysis to evaluate the characteristics of cluster 1, the data was coded as 1 and clusters other than 1 were coded as 0 (reference category) and entered the dependent variable. A stepwise method was applied to the multiple logistic regression analysis in this study to identify independent variables to be retained or removed from the model based on predefined statistical criteria influenced by the inherent characteristics of the sample being analyzed [26]. Collinearity was tested using the Variance Inflation Factor (VIF). As the VIF values for all covariates were small (< 5), no evidence of multicollinearity was found. Bonferroni-Holm correction was applied to adjust for multiple tests [27].

All statistical analyses were performed using SPSS for Windows (version 29; SPSS Inc.). The tests were two-tailed, and the results were considered statistically significant at *p* <  0.05.

The validity of the sample size for factor analysis in this study has been confirmed by previous studies. Vergouwe et al. [28] have suggested a minimum of 100 events and 100 nonevents as external validation samples for a logistic regression analysis with adequate power. Further, a power analysis calculation indicated that for an effect size of 0.5 (Kruskal–Wallis test) and 0.3 (chi-square test) and a power of 0.8, four groups with at least 128 and 210 participants for Kruskal–Wallis and chi-square tests, respectively, would be required.

## Results

### Participants’ characteristics

The study sample comprised 842 men (49.3%) and 865 women (50.7%) (Table 2). Median (25th, 75th percentiles) age and BMI were 40 (30, 50) years and 21.2 (19.2, 23.7), respectively. More than half of the participants lived with their family members from other generations, such as parents or children (55.7%).

**Table 2:** Differences of characteristics of the study participants by each cluster (n = 1,707)

	Total		LPP*HPC (n = 319, 18.7%)		MPP*HPC (n = 400, 23.4%)		MPP*LPC (n=593, 34.7%)		HPP*LPC (n = 395, 23.1%)		*p* value
	n/Median	%/ 25^th^, 75^th^ percentile	n/Median	%/ 25^th^, 75^th^ percentile	n/Median	%/ 25^th^, 75^th^ percentile	n/Median	%/ 25^th^, 75^th^ percentile	n/Median	%/ 25^th^, 75^th^ percentile	
*Demographic and anthropometric variables*											
Age (year)	40	30, 50	38^hi^	29, 48	38^j^	29, 48	42^hj^	32, 52	41^i^	31, 51	< 0.001^m^
*Sex*											
Male	842	49.3	217	68.0	262	65.5	225	37.9	138	34.9	< 0.001^n^
Female	865	50.7	102	32.0	138	34.5	368	62.1	257	65.1	
Body mass index	21.2	19.2, 23.7	22.2^hi^	19.7, 25.0	21.5^jk^	19.9, 24.2	21^hjl^	19.1, 23.5	20.4^ikl^	18.5, 22.5	< 0.001^m^
< 18.5	279	16.3	43	13.5	36	9.0	100	16.9	100	25.3	< 0.001^n^
18.5–25.0	1131	66.3	195	62.1	282	70.5	401	67.6	250	63.3	
> 25.0	297	17.4	78	24.5	82	20.5	92	15.5	45	11.4	
Living status ^a^	2	1, 2	2^d^	0, 2	2	1, 2	2^d^	1, 2	2	1, 2	0.04^m^
*Household income [JPY (USD)]*											
< 2,000,000 (15,000)	213	12.5	39	12.2	46	11.5	74	12.5	54	13.7	0.96^n^
2,000,000–4,000,000 (15,000–30,000)	356	20.9	77	24.1	88	22.0	121	20.4	70	17.7	
4,000,000–6,000,000 (30,000–45,000)	426	25.0	80	25.1	101	25.3	147	24.8	98	24.8	
6,000,000–8,000,000 (45,000–60,000)	278	16.3	48	15.0	66	16.5	99	16.7	65	16.5	
8,000,000–10,000,000 (60,000–75,000)	198	11.6	32	10.0	47	11.8	72	12.1	47	11.9	
> 10,000,000 (75,000)	236	13.8	43	13.5	52	13.0	80	13.5	61	15.4	
*Education*											
Elementary or/junior high school	40	2.3	6	1.9	11	2.8	14	2.4	9	2.3	0.08^n^
High school	409	24.0	85	26.6	88	22.0	149	25.1	87	22.0	
Junior college or vocational school	334	19.6	56	17.6	61	15.3	134	22.6	83	21.0	
College, university or graduate school	924	54.1	172	53.9	240	60.0	296	49.9	216	54.7	
*Psychological variables (range)*											
Interest in health (12–48) ^b^	33	29, 36	29^hij^	25, 34	33^ik^	29, 36	33^hl^	29, 36	35^jkl^	31, 39	<0.001^m^
Attitude toward avoiding food waste (3–18)^c^	14	12, 16	14^h^	12, 16	14^i^	12, 16	14^j^	12, 16	15^hij^	13, 17	<0.001^m^
Gratitude for food (5–20)^d^	15	11, 15	13^hij^	10, 15	15^ik^	12, 15	14^hl^	11, 15	15^jkl^	13, 17	<0.001^m^
*Lifestyle-related variables*											
Frequency of eating out^e^	2	1, 3	2^h^	1, 3	2^i^	1, 3	2^i^	1, 2	2^h^	1, 2	< 0.001^m^
Frequency of home-meal replacement/prepared meal^e^	2	2, 3	3	1, 3	2	2, 3	2	1, 3	3	2, 3	0.14^n^
Frequency of eating together^e^	5	2, 6	4^hi^	1, 5	4	2, 5	5^h^	3, 6	5^i^	3, 6	< 0.001^m^
Frequency of cooking^f^	5	2, 7	4^hi^	2, 6	4^j^	2, 6	5^hk^	2, 7	6^ijk^	4, 7	< 0.001^m^
Recognition of the need to control portion size (4–24)	13	10, 15	11^hij^	9, 13	13^hk^	12, 16	13^ikl^	11, 15	13^jl^	10, 16	< 0.001^m^
Eating out^g^	3	2, 4	3^hi^	2, 5	4^jk^	3, 4	3^hj^	2, 4	3^ik^	2, 5	< 0.001^m^
Cooking^g^	3	3, 4	3^hij^	2, 4	4^h^	3, 4	4^i^	3, 4	4^j^	2, 4	< 0.001^m^
Prepared food^g^	3	2, 4	2^hij^	1, 3	3^h^	2, 4	3^i^	3, 4	3^j^	2, 4	< 0.001^m^
Prepared meal by family member^g^	3	2, 4	2^hij^	1, 3	3^h^	2, 4	3^i^	3, 4	3^j^	2, 4	< 0.001^m^
Physical activity level (METs/week)^d^	792	0, 2079	742^h^	0, 1950	972^i^	107, 2555	594^ij^	0, 1823	990^hj^	132, 2385	< 0.001^m^

LPP*HPC: Low pre-meal portion planning to prevent overeating * low plate clearing behaviors; MPP*HPC: Moderate pre-meal portion planning to prevent overeating * high plate clearing behaviors; MPP*LPC: moderate pre-meal portion planning to prevent overeating * low plate clearing behaviors; HPP*LPC: high pre-meal portion planning to prevent overeating * high plate clearing behaviors

^a^ 0: living alone; 3: three-generation family

^b^Interest in Health Scale [23]. Higher score represents higher interest in health

^c^ Items used in previous study [24]. Higher score represents higher attitude toward avoiding food waste

^d^ Gratitude for food scale for adults (GFS-A) [16]. Higher score represents higher gratitude for food

^e^1: once in a month or less–6: twice in a day or more

^f^1: not at all–8: three times in a day or more

^g^1: Not at all –6: always.Letters (h–l) represent significant statistical differences between each group by using the Bonferroni’s multiple comparison test (adjusted *p* <  0.05). For example, LPP*HPC showed significant differences in age from MPP*LPC (h) and HPP*LPC (i) as represented by their distinct letter assignments.

^m^Kruskal-Wallis test

^n^ Chi-square test

### Cluster description

**Fig. 1:**
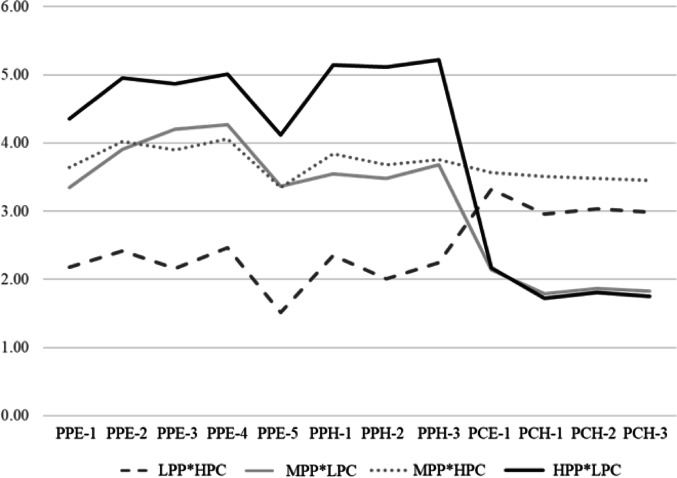
Pre-meal portion planning to prevent overeating and plate clearing behaviors by each cluster (n = 1,707)PPE: Pre-meal portion planning to prevent overeating when eating out; PPH: Pre-meal portion planning to prevent overeating at home; HPP: high frequency of pre-meal portion planning to prevent overeating; HPC: high frequency of plate clearing behaviors; LPP: low frequency of pre-meal portion planning to prevent overeating; LPC: low frequency of plate clearing behaviors; MPP: moderate frequency of pre-meal portion planning to prevent overeating; PCE: plate clearing behavior when eating out; PCH: plate clearing behavior at home.PCE1-5, PCH1-3: 1: Not at all − 6: alwaysPCE1, PCH1-3: 1: I do not eat (leave/take home) more than I can eat without difficulty on the spot, regardless of how much the menu offers - 6: I eat more than twice as much food as I can eat withoutdifficulty

The cluster analysis identified four groups. Figure 1 shows the mean frequencies of PPP-O and PCBs for each cluster. These behavioral patterns were referred to as follows: low frequency of PPP-O and high PCBs (LPP*HPC; n = 319, 18.7%), moderate frequency of PPP-O and high PCBs (MPP*HPC; n = 400, 23.4%), moderate frequency of PPP-O and low PCBs (MPP*LPC; n = 593, 34.7%), and high frequency of PPP-O and low PCBs (HPP*LPC; n = 395, 23.1%).

### Differences in participants’ characteristics by cluster

**Fig. 2:**
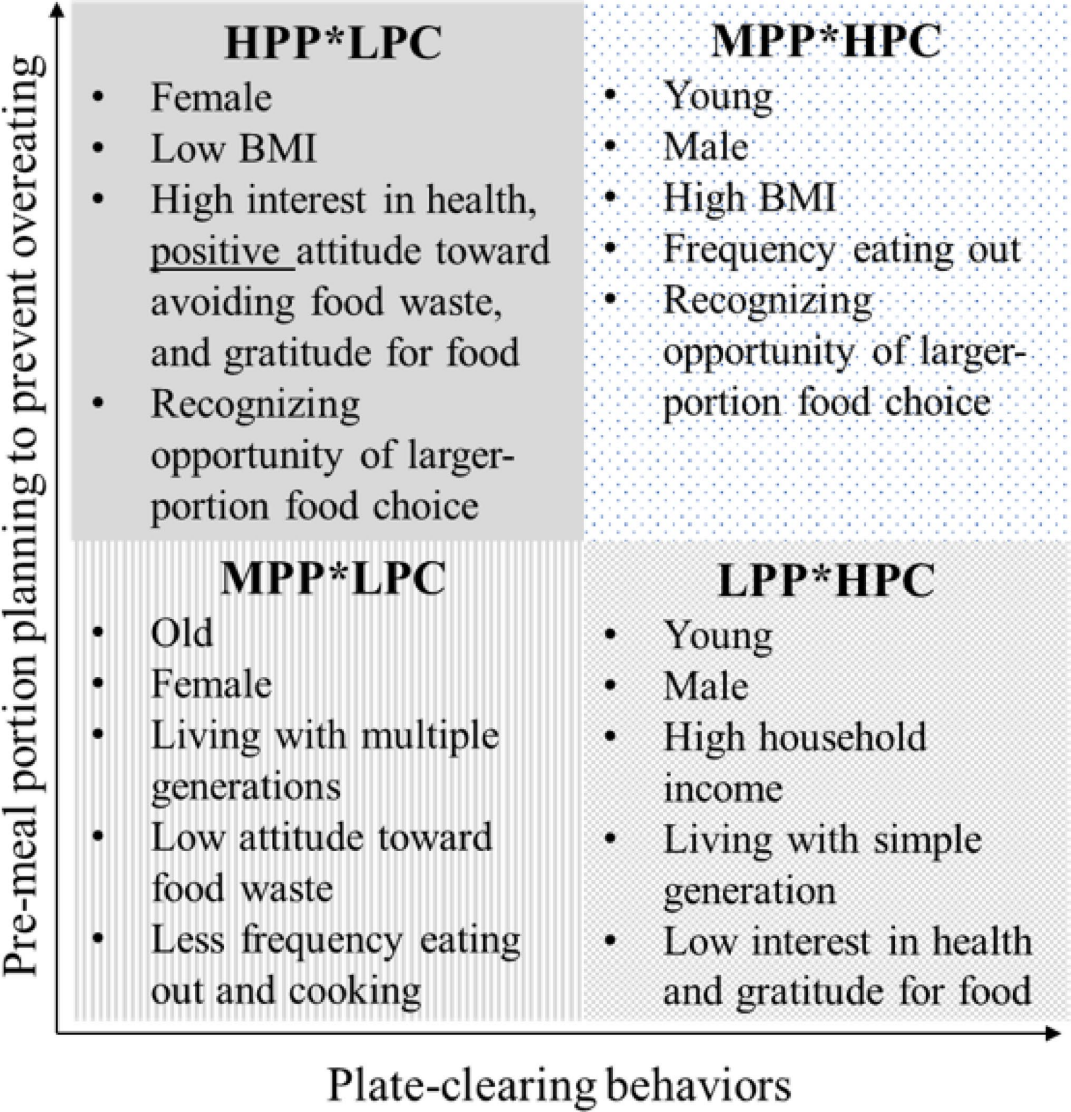
Schematic diagram of differences between clustersPPE: Pre-meal portion planning to prevent overeating when eating out; PPH: Pre- meal portion planning to prevent overeating at home; HPP: high frequency of pre- meal portion planning to prevent overeating; HPC: high frequency of plate clearing behaviors; LPP: low frequency of pre-meal portion planning to prevent overeating; LPC: low frequency of plate clearing behaviors; MPP: moderate frequency of pre-meal portion planning to prevent overeating

Table 2 and Fig. 2 show the differences in the characteristics of the study participants, including demographic and anthropometric, psychological, and lifestyle-related variables, for each cluster. Almost all variables differed for each cluster, except for household income, education, and frequency of prepared meals (*p* =  0.961, 0.082, and 0.137, respectively). More than 60% of the participants were men for LPP*HPC (68.0%) and MPP*HPC (65.5%), while most were women for MPP*LPC (62.1%) and HPP*LPC (65.1%). The proportion of people with obesity and overweight (BMI > 25.0) was the largest in the LPP*HPC cluster (24.5%) and the lowest in the HPP*LPC cluster (11.4%), whereas the proportion of underweight (BMI < 18.5) participants was the largest in the HPP*LPC cluster (25.3%) and the lowest in the LPP*HPC cluster (13.5%). All three psychological variables, such as interest in health, attitude toward avoiding food waste, and gratitude for food, differed by cluster, and the total scores were the highest for HPP*LPC and the lowest for LPP*HPC (*p* <  0.001). Participants in the HPP*LPC cluster cooked most frequently out of the four clusters (median =  6: “once a day”;* p* <  0.001). Participants in the LPP*HPC cluster did not recognize the need to control portion size in various eating scenarios, such as eating out, home cooking, eating prepared foods, and eating meals prepared by family members (*p* <  0.001).

### Odds ratios of cluster allocation based on participants’ demographic, anthropometric, psychological, and lifestyle-related variables

The ORs of cluster allocation based on the participants’ demographic, anthropometric, psychological, and lifestyle-related variables are described in Table 3. Being young [OR (95% CI): 0.98 (0.97–0.996), *p* =  0.01], male [0.42 (0.32–0.56), *p* < 0.001], having a high household income [1.10 (1.01–1.21), *p* =  0.03], living with simple generations, such as living alone and one generation family [0.80 (0.69–0.94), *p* = 0.01], and having low scores of interest in health [0.91 (0.89–0.94), *p* <  0.001] and gratitude for food [0.92 (0.89–0.96), *p* <  0.001] were associated with LPP*HPC cluster. Individuals were more likely to be in the MPP*HPC cluster if they were young [0.99 (0.98-–0.996), *p* =  0.01], male [0.45 (0.36–0.58), *p* <  0.001], had a high BMI (<  18.5) [0.55 (0.37–0.81), *p* =  0.002], frequently ate out [1.19 (1.05–1.35), *p* =  0.01], and realized needs for adequate amount of food choice [1.06 (1.03–1.10), *p* <  0.001]. Those belonging to the MPP*LPC cluster were more likely to be older [1.02 (1.01–1.03), *p* <  0.001], female [2.58 (2.02–3.28),* p* <  0.001], live with multiple generations [1.19 (1.05–1.35), *p* =  0.01], have low scores of attitude toward of food waste [0.91 (0.87–0.94), *p* <  0.001], and less frequently eat out [0.88 (0.78–0.99), *p* =  0.03] and cook [0.92 (0.88–0.97), *p* =  0.002]. Finally, individuals belonging to the HPP*LPC cluster were more likely to be female [1.55 (1.18–2.04), *p* =  0.002]; have lower BMIs (<  18.5) [1.74 (1.27–2.37), *p* < 0.001]; have higher interest in health scores [1.04 (1.02–1.07), *p* <  0.001] and gratitude for food [1.11 (1.06–1.15), *p* < 0.001]; have a more positive attitude toward avoiding food waste [1.13 (1.08–1.19), *p* <  0.001]; and recognize the needs for control portion size [1.05 (1.01–1.08), *p* = 0.01].

**Table 3:** Odds ratios for cluster allocation based on participants’ demographic, anthropometric, psychological, and lifestyle variables^a^

	LPP*HPC (n = 319)			MPP*HPC (n = 400)			MPP*LPC (n = 593)			HPP*LPC (n = 395)		
	OR	95%CI^b^	*p*^c^	OR	95%CI^b^	*p*^c^	OR	95%CI^b^	*p*^c^	OR	95%CI^b^	*p*^c^
*Demographic and anthropometric variables*												
Age (year)	**0.98**	**0.97–0.996**	**0.01**	**0.99**	**0.98–0.996**	**0.01**	**1.02**	**1.01–1.03**	**< 0.001**	–		
*Gender*												
Male	1			1			1			1		
Female	**0.42**	**0.32–0.56**	**<0.001**	**0.45**	**0.36–0.58**	**< 0.001**	**2.58**	**2.02–3.28**	**< 0.001**	**1.55**	**1.18–2.04**	**0.002**
Body mass index						**0.01**						**< 0.001**
< 18.5	–			**0.55**	**0.37–0.81**	**0.002**	–			**1.74**	**1.27–2.37**	**< 0.001**
18.5–25.0				1						1		
> 25.0				1.04	0.77–1.41	0.79				0.79	0.55–1.15	0.22
Living status (0: living alone; 3: three-generation family)	**0.80**	**0.69–0.94**	**0.01**	–			**1.19**	**1.05–1.35**	**0.01**	–		
Household income (JPY)	**1.10**	**1.01–1.21**	**0.03**	–			–			–		
Education	–			–			–			–		
*Psychological variables*												
Interest in health (12–48)	**0.91**	**0.89–0.94**	**<0.001**	–			-			**1.04**	**1.02–1.07**	**< 0.001**
Attitude toward avoiding food waste (3–18)	–			–			**0.91**	**0.87–0.94**	**< 0.001**	**1.13**	**1.08–1.19**	**< 0.001**
Gratitude for food (5–20)	**0.92**	**0.89–0.96**	**<0.001**	–			–			**1.11**	**1.06–1.15**	**< 0.001**
*Lifestyle-related variables*												
Frequency of eating out (1: once in a month or less–6: twice in a day or more)	–			**1.19**	**1.05–1.35**	**0.01**	**0.88**	**0.78–0.99**	**0.03**	–		
Frequency of home-meal replacement/prepared meal (1: once in a month or less–6: twice in a day or more)	–			–			–			–		
Frequency of eating together (1: once in a month or less–6: twice in a day or more)	–			–			–			–		
Frequency of cooking (1: not at all–8: 3 times in a day or more)	–			–			**0.92**	**0.88–0.97**	**0.002**	1.07	1.01–1.14	0.03
Recognition of the need to control portion size (4–24)	**0.88**	**0.85–0.92**	**< 0.001**	**1.06**	**.03–1.10**	**< 0.001**	–			**1.05**	**1.01–1.08**	**0.01**
Physical activity level (METs/week)	–			–			–			–		
Adjusted R^2^	0.21			0.09			0.09			0.17		

HPP: high frequency of pre-meal portion planning to prevent overeating; HPC: high frequency of plate clearing behaviors; LPP: low frequency of pre-meal portion planning to prevent overeating; LPC: low frequency of plate clearing behaviors; MPP: moderate frequency of pre-meal portion planning to prevent overeating; OR, odds ratio; CI, confidence index

^a^Four multiple logistic regression analysis (stepwise method) were conducted. Simple logistic regression analysis was omitted. In column LPP*HPC, dependent variables are LPP*HPC (1) and other clusters (0; reference category)

^b^Cells with a hyphen indicate that they were not entered into the regression equation by the Stepwise method

^c^Significant differences by Bonferoni-Holm correction, which is the method to counteract the problem of multiple comparisons, are highlighted bold (27; Holm, 1979)

## Discussion

In this study, four clustering patterns concerning PPP-O and PCBs and their independent predictors were identified: LPP*HPC, MPP*HPC, MPP*LPC, and HPP*LPC. The LPP*HPC cluster that associates being younger, male, and with less frequent recognition of the need for portion control had the highest proportion of people with obesity among the four clusters. Psychological factors, such as interest in health, positive attitude toward avoiding food waste, and gratitude for food, were described as independent predictors of several clusters. The results support the possibility of a trade-off between food waste and weight gain.

Participants in the HPP*LPC group were more likely to be female, have a BMI of less than 18.5, and have a higher levels of health concern and attitude toward avoiding food waste and gratitude for food. This result is consistent with the results of a previous study that reported the characteristics of those who order an appropriate amount of food, such as being female and having a high subjective health status [20]. Although this cluster was expected to have a sustainable and healthy pattern that did not cause a trade–off between weight gain and food waste due to having the highest frequency of PPP-O of the four clusters, a significantly higher proportion had a BMI below 18.5, which is an unhealthy outcome. In Japan, thinness is serious problem, often caused by undernutrition, and raises future health risks; moreover, obesity and the prevalence of thinness is particularly high among young women [29, 30]. Some of the participants in the HPP*LPC group may have had incorrect perceptions of their own portion size to maintain their health. In a previous study, female participants who had a low BMI and higher scores for restrained eating tended to estimate lower portion sizes [31]. Although few studies examining the relationship between portion size estimation and BMI among the Japanese population have been conducted, it is possible that the participants in the present study implemented PPP-O based on false perceptions of their own adequacy to select portion sizes to manage their health. In this context, the results for the Japanese population in the present study suggest the need to ensure that education to promote PPP-O does not exacerbate the risk of eating disorders and over-restriction. Therefore, the results suggest that to achieve both weight control and food waste reduction, it is necessary to promote education to correctly recognize the appropriate amount of food for oneself, as well as to promote PPP-O and PCBs from the perspective of improving individual and global health.

Our result that the LPP*HPC cluster, which had a lower frequency of PPP-O and a higher frequency of PCBs the LPP*HPC cluster, had the highest percentage of people with obesity persons among the four clusters could be one basis for the hypothesis of this study. Our hypothesis is that large portion size selection creates a trade-off between weight gain and food waste and may lead to weight gain in those who choose PCBs. Thus, in this cluster, PCBs may function as maladaptive behaviors owing to the preference for large portion sizes. Being younger, male, and feeling the need for PPP-O less frequently were independently associated with the LPP*HPC cluster. It is reasonable to assume that the frequency of PPP-O is naturally low if the frequency of feeling the need for PPP-O is also low. However, in a previous study describing the serving sizes of fixed meal offerings in the restaurant industry, many of the target meals exceeded the Japanese standard for the portion-controlled food in a meal for an adult (450–650 or 650–850 kcal by Smart meal) [32, 33]. This means that participants in this cluster may also be exposed to a food environment that impedes PPP-O. To make PPP-O easier for individuals, the restaurant industry is encouraged to modify the food environment, for example through serving meals with recommended portion sizes and flexible adaptations to portion size customization, which previous studies have pointed to as a factor promoting healthy eating behaviors [34, 35]. It may lead to reducing food waste and making plate clearing less maladaptive.

Participants classified in the MPP*LPC cluster, which had the highest number of individuals, were expected to generate food waste due to leftovers because of their moderate and low frequency of PPP-O and PCBs, respectively. The results of the low percentage of those with a BMI of 25.0 or higher and low scores for attitude toward food waste support the possibility that moderate PPP-O scores result in a trade-off between food waste and weight gain and thus food waste was chosen. Conversely, the MPP*HPC cluster had a higher percentage of those with a BMI of 25.0 or higher compared to the MPP*LPC cluster, which may have led to the selection of weight gain due to the above trade-off. The behavioral patterns identified by the present study suggest the importance of education to promote PPP-O to avoid making the trade-off between weight gain and food waste.

Psychological factors such as interest in health, attitude toward avoiding food waste, and gratitude for food were independent predictors of several clusters in the present study. The results of this study support the findings of many previous studies that psychological factors are involved in healthy dietary and food waste avoidance behaviors [24, 36–38]. Although a number of previous studies have evaluated the associations of interest in health with health behaviors [39] and food waste concerns with food waste avoidance behaviors [40], few studies have examined these factors simultaneously [2]. The finding that psychological factors concerning both higher health and food waste concerns were independently associated with the HPP*LPC cluster indicates that parallel education on health and food waste avoidance promotion may be necessary to implement sustainable and healthy dietary behaviors without making a tradeoff between poor weight control and increased food waste. In Japan, educational materials have been developed to promote the ordering and consumption of portion-controlled food [41]. Therefore, based on the results of this study, further research should examine the effectiveness of such education.

This study had several limitations. First, several items used in this study, such as PPP-O and PCBs, were not validated. However, both items showed significant correlations with the validated questionnaire items, and test–retest reliability was confirmed in the questionnaire setting. It is necessary to confirm the validity of these questions in the future by comparing them with consumers’ actual behaviors, such as actual food waste behaviors. Cross-cultural comparison of PCBs with the scale of PCBs for individuals from the West, such as the scale developed by Robinson et al., would also be necessary [4]. Research positioning PPP-O as a subset of restrictions is also necessary. Second, since this study focuses on adult Japanese consumers, the results cannot be generalized to other populations. Japanese people traditionally have a social norm of avoiding leaving leftovers owing to their cultural and religious backgrounds [7]; therefore, the characteristics of the patterns of PPP-O and PCBs among Japanese people may differ from the trends shown for countries in previous studies. Third, given that this study focused on food choice, it did not consider the post-meal behaviors that may affect the trade-off between food waste and weight control, such as taking leftovers home with them. Therefore, future studies should account for a series of eating behaviors from food choice to disposal. Fourth, given that participants did not report their food waste behaviors/amount, this study is speculative in nature. Further research examining the association of PPP-O and PCBs with food waste amount is required to strengthen evidence of the trade-off of weight gain and food waste. Finally, the limitation based on the web-research, such as reporting and response bias, was observed in a previous study [16].

Despite these limitations, this study is the first to examine the clustering patterns of PPP-O and PCBs, considering their dietary behavioral scenarios and characteristics. This study contributes to identifying desirable consumer behaviors to maintain physical and planetary health and develop an implementation strategy for PPP-O and PCBs. Further research should validate the scale to measure PPP-O and PCBs among the Japanese population to compare PPP-O and PCBs cross-culturally, and to determine the longitudinal impact of these patterns on the risk of non-communicable disease and environmental outcomes such as BMI and the amount of individual food waste. Furthermore, future research should explore how much of the desire to implement food-choice behaviors results in this happening.

## Conclusion

This study aimed to (1) identify patterns of PPP-O and PCBs in various meal situations and (2) describe the demographic, anthropometric, psychological, and lifestyle-related characteristics of those who follow each pattern. Four clustering patterns concerning PPP-O and PCBs and their independent predictors were identified: (1) LPP*HPC, with predictors of lower age, being male, smaller household size, larger household income, and less interest in health and gratitude for food, and recognizing the need to control portion sizes; (2) MPP*HPC, with predictors of lower age, being male, slightly lower BMI (<  18.5), higher frequency of eating out, and recognizing the need to control portion sizes; (3) MPP*LPC, with predictors of higher age, being female, larger household size, low attitude toward avoiding food waste, and a lower frequency of eating out and cooking; and (4) HPP*LPC, with predictors of being female, higher proportion of lower BMI (< 18.5), high interest in health, attitude toward avoiding food waste, and gratitude for food, and recognizing the need to control portion sizes. This study contributes to identifying desirable consumer behaviors to maintain physical and global health and develop an implementation strategy for PPP-O and PCBs.

## Supplementary Information

Below is the link to the electronic supplementary material.

Supplementary Material 1

Supplementary Material 2

## Data Availability

Data will be made available on reasonable request.
