# Supplementary material for: Are weight control and food waste a trade-off?: A clustering of pre-meal portion planning and plate-clearing behaviors among Japanese adult consumers
Source: Eur J Nutr. 2025 Nov 18;64(8):322. doi: 10.1007/s00394-025-03837-0 (PMC12627122; doi:10.1007/s00394-025-03837-0)
Supplement: Supplementary file 1 — Supplementary Material 1 [file 394_2025_3837_MOESM1_ESM.docx]

Appendix A

**Questionnaires concerning key variables sent to participants in the study**

**Pre-meal portion planning to prevent overeating**

**<Eating out>**

Source: original (based on Nishida, et al. 2023) [1]

Instruction: How often do you usually do the following when ordering a meal for one person at a restaurant?

Options: 1: Not at all, 2: rarely, 3: seldom, 4: sometimes, 5: often, 6: always

PPE-1. I check the serving size of food before entering a restaurant or choose a restaurant where I already know the serving size of food or can adjust the serving size of food.

PPE-2. When I select a menu, I check weather I can eat the food I want to order without difficulty.

PPE-3. When I want to order food with a larger serving size than I can eat without difficulty, I choose a smaller size if the menu has a range of sizes.

PPE-4. When I want to order food with a larger serving size than I can eat without difficulty, I order food with a smaller serving size if available and if I can get a discount by reducing the size.

PPE-5. When I want to order food with a larger serving size than I can eat without difficulty, I ask the waiter for a smaller portion even if the menu does not have a size range.

<Home>

Source: original

Instruction: You are about to have a meal at home. In the following situations, when you feel like there is more food on the table than you can comfortably eat, do you ever try to reduce the amount you can eat? Please answer by thinking about what you do when you put food on your table, regardless of whether you leave it or not. For the question “reduce,” please answer by account for the actions you take before starting to eat, such as freezing/refrigerating food or sharing it with a family member.

Options: 1: Not at all, 2: rarely, 3: seldom, 4: sometimes, 5: often, 6: always

PPH-1. When I feel that the amount of food I prepare is more than I can eat without difficulty, I reduce it to the amount I can eat.

PPH-2. When I feel that the amount of food (e.g. lunch boxes, prepared foods, or instant foods) I purchased is more than I can eat without difficulty, I reduce the amount until I can finish the food.

PPH-3. When the amount of food prepared by family members is more than I can eat without difficulty, I reduce the amount until I can finish the food.

**Plate clearing behavior**

**<Eating out>**

Source: original

Instruction: None.

Options: 1: I do not eat (leave/take home) more than I can eat without difficulty on the spot, regardless of serving size of the food, 2: I eat up to 1.2 times as much food as I can eat without difficulty, 3: I eat up to 1.5 times as much food as I can eat without difficulty, 4: I eat up to twice as much food as I can eat without difficulty, 5: I eat more than twice as much food as I can eat without difficulty

PCE-1. When eating out, if you feel that the amount of food is too much (there is more food in front of you than you can eat without difficulty), do you eat up the meal, even if you have to push yourself?

<Home>

Source: original

Instruction: In the following situations, when there is more food on the table than you can comfortably eat, are you able to eat even if you force yourself a little? Select the answer that most closely resembles your usual behavior.

Options: 1: I do not eat (leave/take home) more than I can eat without difficulty on the spot, regardless of serving size of the food, 2: I eat up to 1.2 times as much food as I can eat without difficulty, 3: I eat up to 1.5 times as much food as I can eat without difficulty, 4: I eat up to twice as much food as I can eat without difficulty, 5: I eat more than twice as much food as I can eat without difficulty

PCH-1. When I cook and prepare (serve) a larger amount of food than I can eat without difficulty, …

PCH-2. When I feel that the amount of food I purchased (e.g., lunchbox and prepared food) or instant food I cooked is more than I can eat without difficulty, …

PCH-3. When the amount of food prepared by my family members was more than I could reasonably eat, …

**Recognition of the need to control portion size**

Source: original

Instruction: How often do you feel the following in your daily life? Please select the one that best applies to you.

Option: 1: Not at all, 2: rarely, 3: seldom, 4: sometimes, 5: often, 6: always

Items:

1. When eat out, I sometimes feel like ordering a menu item with a larger portion size than I can comfortably eat.

2. When cooking, I sometimes feel that the amount of food prepared is more than I (we) can comfortably eat.

3. I sometimes feel that the amount of prepared foods that I buy is more than I can comfortably eat.

4. I sometimes feel that the amount of food prepared by my family is more than I can comfortably eat.

**Interest in Health**

Source: Ozawa, et al. 2021 [2]

Instruction: What do you think about the following your thoughts on health? Please select the one that best applies to you.

Option: 1: Disagree, 2: somewhat disagree, 3: somewhat agree, 4: agree

Items:

(1) I’m very self-conscious about my health.

(2) I’m interested in information about my health.

(3) I pay attention to changes in my health condition.

(4) I am more health conscious than people around me.

(5) I am willing to spend some extra money for my health.

(6) I do everything I can to stay healthy.

(7) We should spend some extra time for health.

(8) I want to put health first in my living.

(9) Work and income are more important than health.

(10) I worry about my health only when I get sick.

(11) Hobbies and leisure activities are more important than health.

(12) Rather than prevent illness, I just want to be cured when I get sick.

**Attitude toward food waste**

Source: Stancu, et al. 2016^a^ [3]

Instruction: To what extent do the following feelings regarding eating behaviors apply to you? Please select the one that best applies to you.

Option: 1: Strongly disagree, 2: disagree, 3: somewhat disagree, 4: slightly agree, 5: agree, 6: strongly agree

Items:

1. In my opinion, wasting food is extremely negative.

2. In my opinion, wasting food is extremely foolish.

3. In my opinion loading the environment with my household’s food waste is extremely negative.

^a^ In this study, the questionnaire was based on Stancu et al.’s questionnaire but was revised to fit other questionnaires.

**Gratitude for food**

Source: Kawasaki, et al. 2024 [4]

Instruction: Please select the answer that best describes your everyday behavior and feelings.

Option: 1: Strongly disagree, 2: disagree, 3: agree, 4: strongly agree

Items:

1. When I eat food, I feel gratitude toward the food which gives me life.

2. When I eat food, I feel gratitude toward the people who transported the food.

3. When I eat food, I feel gratitude toward the fact that my meals exist thanks to many people’s efforts.

4. When I eat food, I feel gratitude toward the food producers.

5. When I eat food, I feel gratitude toward the people who cooked my meals.

**Reference**

1. Nishida I, Akamatsu R, Tonsho N (2023) Characteristics of people who order the appropriate amount of food at restaurants. Japanese J Nutr Diet 81:68–74. https://doi.org/10.5264/EIYOGAKUZASHI.81.68

2. Ozawa C, Ishikawa H, Kato M, Fukuda Y (2021) Development of the Interest in Health Scale to understand the “population indifferent to health.” Japanese J Heal Educ Promot 29:266–277. https://doi.org/10.11260/KENKOKYOIKU.29.266

3. Stancu V, Haugaard P, Lähteenmäki L (2016) Determinants of consumer food waste behaviour: Two routes to food waste. Appetite 96:7–17. https://doi.org/10.1016/j.appet.2015.08.025

4. Kawasaki Y, Nagao-Sato S, Shimpo M, et al (2024) Development and validation of the gratitude for food scale for Japanese adults. Glob Health Promot ahead-of-print. https://doi.org/10.1177/17579759241235890
