# Supplementary material for: Are weight control and food waste a trade-off?: A clustering of pre-meal portion planning and plate-clearing behaviors among Japanese adult consumers
Source: Eur J Nutr. 2025 Nov 18;64(8):322. doi: 10.1007/s00394-025-03837-0 (PMC12627122; doi:10.1007/s00394-025-03837-0)
Supplement: Supplementary file 2 — Supplementary Material 2 [file 394_2025_3837_MOESM2_ESM.docx]

Appendix B

**The original Japanese version of questionnaires developed for the survey**

**Pre-meal portion planning to prevent overeating**

**<Eating out>**

Instruction:ふだん，飲食店で1人分の食事を注文するとき，あなたは次の行動をどのくらいの頻度で行いますか

Options: 1:全くしない, 2: ほとんどしない, 3: あまりしない, 4: たまにする, 5: ときどきする, 6: いつもする

Items:

PPE-1. 入店前に提供量を確認したり，提供量が分かっているお店や量を調節できるお店を選ぶ。

PPE-2. メニューを選ぶ際に，頼みたいメニューが、自分が無理なく食べきれる量かどうか確認する。

PPE-3. 自分が無理なく食べきれる量より提供量が多いメニューを頼みたい時、メニューのサイズ展開があれば，小さいサイズを選ぶ。

PPE-4. 自分が無理なく食べきれる量より提供量が多いメニューを頼みたい時、メニューのサイズ展開があり、サイズダウンにより値引きがされるなら，サイズを下げる。

PPE-5. 自分が無理なく食べきれる量より提供量が多いメニューを頼みたい時、メニューのサイズ展開がなくても、お店の人に少なめをお願いする。

<Home>

Instruction: あなたはこれからご自宅で食事します。次のような場面で、自分の適量（無理なく食べきれる量）よりも多くの量が食卓に並びそうな時、無理なく食べきれる量まで減らすことはありますか。食べ残す・食べ残さないに関係なく、あなたが食べる物を、自分の食卓に載せる時の行動を思い浮かべてお答えください。質問項目の「減らす」は、冷凍/冷蔵保存する、家族に食べてもらうなど、食べ始める前に行う行動を総合してお答えください。

Options: 1:全くしない, 2: ほとんどしない, 3: あまりしない, 4: たまにする, 5: ときどきする, 6: いつもする

Items:

PPH-1. 自分で調理した料理が、自分が無理なく食べきれる量よりも多く出来上がりそうな時に、食べきれる量まで減らす。

PPH-2. 購入したお弁当やお惣菜、調理したインスタント食品が、自分が無理なく食べきれる量より多いと感じた時は、食べきれる量まで減らす。

PPH-3. 購入したお弁当やお惣菜、調理したインスタント食品が、自分が無理なく食べきれる量より多いと感じた時は、食べきれる量まで減らす。

**Plate clearing behavior**

**<Eating out>**

Instruction: PCE-1. 外食の際、「量が多い（目の前に、自分が無理なく食べきれる量よりたくさんの食べ物がある）」と感じた時、あなたは、多少無理してでも、食事を食べきりますか。普段のあなたの行動に最も近いものを一つ選択してください。

Options:

1.あなたが無理なく食べきれる量の2倍以上の量でも、食べきる。

2. あなたが無理なく食べきれる量の2倍量までなら、食べきる。

3. あなたが無理なく食べきれる量の1.5倍量までなら、食べきる。

4. あなたが無理なく食べきれる量の1.2倍量までなら、食べきる。

5. あなたが無理なく食べきれる量より多い分は、メニューの提供量にかかわらず、その場で食べない（食べ残す/持ち帰る）

<Home>

Instruction: 次のような場面で、あなたの適量（無理なく食べきれる量）よりも多くの量が食卓に並んでいる時、あなたは、多少無理してでも、食事を食べきりますか。普段のあなたの行動に最も近いものを選択してください。

Options:

1. 無理なく食べきれる量の2倍以上の量でも、食べきる。

2. 無理なく食べきれる量の2倍量までなら、食べきる。

3. 無理なく食べきれる量の1.5倍量までなら、食べきる。

4. 無理なく食べきれる量の1.2倍量までなら、食べきる。

5. 無理なく食べきれる量より多い分は、メニューの提供量にかかわらず、その場で食べない（食べ残す/持ち帰る）

Items:

PCH-1. 自分で調理をしていて、自分が無理なく食べきれる量よりも多く、料理が出来上がって（盛り付けて）しまった時。

PCH-2. 購入したお弁当やお惣菜、調理したインスタント食品が、自分が無理なく食べきれる量より多いと感じた時。

PCH-3. 家族が盛り付けた食べ物が、自分が無理なく食べきれる量よりも多かった時。

**Recognition of the need to control portion size**

Instructions: あなたは普段、次のように感じる頻度はどのくらいありますか。最も当てはまるもの一つを選択してください。

Option: 1:全くない, 2: ほとんどない, 3: あまりない, 4: たまにある, 5: ときどきある, 6: いつもある

Items:

1. 外食の際に、自分が無理なく食べきれる量より提供量が多いメニューを頼みたいと感じる。

2. 調理する際、自分(達)が無理なく食べきれる量よりも多く料理が出来上がってしまったと感じる。

3. 購入したお弁当やお惣菜、調理したインスタント食品が、自分が無理なく食べきれる量より多いと感じる。

4. 家族が盛り付けた食べ物が、自分が無理なく食べきれる量よりも多いと感じる。
